# A Retrospective Study on Prognostic Factors and Systemic Treatments of Refractory Meningiomas

**DOI:** 10.3390/curroncol32090516

**Published:** 2025-09-16

**Authors:** Dan-Thanh Christine Nguyen, Cyril Nader, Karl Bélanger, Sarah Lapointe, Bernard Lemieux, Émilie Lemieux-Blanchard, Jean-Paul Bahary, Laura Masucci, Carole Lambert, David Roberge, Robert Moumdjian, Moujahed Labidi, Romain Cayrol, Marie Florescu

**Affiliations:** 1 Department of Hematology-Oncology, Centre Hospitalier de l’Université de Montréal, Montréal, QC H2X 1R6, Canada; karl.belanger.med@ssss.gouv.qc.ca (K.B.); sarah.lapointe.med@ssss.gouv.qc.ca (S.L.); bernard.lemieux.med@ssss.gouv.qc.ca (B.L.); emilie.lemieux-blanchard.med@ssss.gouv.qc.ca (É.L.-B.); marie.florescu.med@ssss.gouv.qc.ca (M.F.); 2 Department of Anesthesia, Centre Hospitalier de l’Université de Montréal, Montréal, QC H2X 1R6, Canada; cyril.nader.med@ssss.gouv.qc.ca; 3 Department of Radio-Oncology, Centre Hospitalier de l’Université de Montréal, Montréal, QC H2X 1R6, Canada; paul.bahary.med@ssss.gouv.qc.ca (J.-P.B.); giuseppina.laura.masucci.med@ssss.gouv.qc.ca (L.M.); carole.lambert.med@ssss.gouv.qc.ca (C.L.); david.roberge.med@ssss.gouv.qc.ca (D.R.); 4 Department of Neurosurgery, Centre Hospitalier de l’Université de Montréal, Montréal, QC H2X 1R6, Canada; robert.a.moumdjian.med@ssss.gouv.qc.ca (R.M.); moujahed.labidi.med@ssss.gouv.qc.ca (M.L.); 5 Department of Pathology, Centre Hospitalier de l’Université de Montréal, Montréal, QC H2X 1R6, Canada; romain.cayrol@umontreal.ca

**Keywords:** meningioma, systemic treatments, prognostic factor, bevacizumab

## Abstract

Standard systemic treatment has not been established for refractory meningioma. This retrospective study aimed to identify prognostic factors for overall survival and document outcomes of systemic therapies. We reviewed patients with meningioma followed at CHUM hospital between 2006 and 2022. Only patients with progression after first-line treatment were included. Among 750 patients, 107 (14%) experienced progression after first-line treatment. They were divided into two groups: Group 1 (*n* = 69, 64%) received salvage local treatments, and Group 2 (*n* = 38, 36%) received additional salvage systemic treatments. The median follow-up time from diagnosis was 7.5 years. 10-year OS was 88.3% (Group 1) vs. 67.2% (Group 2) (*p* = 0.009). Mean survival after stopping systemic treatment was 8.94 months. Key prognostic factors for poorer survival included age ≥ 65 (HR = 2.82; *p* = 0.009), WHO grade 2 or 3 (HR = 4.25; *p* = 0.004), and progression after second-line treatment (HR = 4.77; *p* = 0.004). Bevacizumab was associated with a mPFS of 12 months and 1-year OS of 64,6%, whereas non-Bevacizumab treatments—including Hydroxyurea, Somatostatin, and Sunitinib—were associated with a mPFS of 7 months and 1-year OS of 52,6%. This study highlights the fatal nature of recurrent meningiomas and the urgent need for systemic treatments that can improve their survival.

## Simple Summary

Meningiomas are the most common type of brain tumor. While most can be treated with surgery and radiotherapy, some tumors return and no longer respond to these treatments. Few studies have examined the evolution of patients with recurrent meningioma and their treatments. Our study identified signs that may help physicians recognize these patients earlier and described their outcomes when receiving additional medical treatments. We found that recurrent meningiomas can be life-threatening. Among the treatments studied, Bevacizumab appeared to provide benefits and may be a reasonable option when surgery and radiotherapy no longer work. However, there remains an urgent need to develop better treatments for this group of patients. Our findings provide useful information to help guide physicians and support further research to improve care and survival for people living with recurrent meningioma.

## 1. Introduction

Meningiomas represent the most common primary brain tumors, representing approximately 40% of all central nervous system neoplasms from 2016 to 2020. Their incidence increases with age, particularly affecting individuals over 40 years old, with higher rates observed in black populations (IRR: 1.20–1.31) and females (IRR: 1.74–3.59) [1,2]. A significant risk factor for meningioma development is prior cranial radiotherapy, with a relative risk (RR) ranging from 6 to 10. Additionally, individuals with hereditary neurofibromatosis type 2 (NF2) face up to a 50% likelihood of developing meningiomas, often with multiple tumor formations [3].

Although most meningiomas are benign, 20% are classified as high-grade (WHO grade 2–3) and carry a higher risk of recurrence (OR: 13.83) [1,4]. Key prognostic factors linked to poorer outcomes include age over 40 years (HR: 5.64) and male sex (HR: 1.44) [1].

While surgical resection and radiotherapy remain the standard treatments for symptomatic or progressive meningiomas, no established systemic therapy exists for refractory cases that progress despite these interventions [5]. In 2021, the European Association of Neuro-Oncology (EANO) issued a Level C recommendation for Bevacizumab in cases where no other local treatment options are feasible. However, this recommendation is based on limited evidence [6].

Identifying prognostic factors for survival and assessing the efficacy of systemic treatments in these patients is crucial to improving management strategies. Thus, this retrospective study aims to identify prognostic factors for overall survival in refractory meningioma and describe patient evolution with systemic treatments.

## 2. Materials and Methods

### 2.1. Study Design

This single-center retrospective study was conducted among patients with refractory meningioma at the Montreal University Hospital Center (CHUM-Centre Hospitalier Universitaire de Montréal). Patients who had a first follow-up for meningioma management between January 2006 and December 2022 were identified through a local registry, SARDO, and through the database of the radio-oncology department. Patients’ records were then reviewed through electronic files (OACIS) from the CHUM until February 2025. Ethical approval was received from the local institutional review board.

### 2.2. Patients

Patients either had a radiological or pathologic diagnosis of meningioma. All patients over 18 years old with radiological progression of meningioma after a first-line treatment were included. Radiological progression was defined according to RANO criteria [7]. A global reduction between 1% and 50% was classified as a minor response in our study.

Patients who did not receive initial treatment and those who did not show progression after first-line treatment were excluded, as well as those with less than 6 months of follow-up at CHUM.

All patients received surgery and/or radiotherapy as first-line treatment. After progression, the population was divided into two groups. Group 1 received salvage local treatments such as surgery and/or radiotherapy for progression. Group 2 received additional salvage systemic treatments for progression after being refractory to local treatments.

The following demographic and tumor data were collected: age, sex, personal and family history of cancer, NF2 mutation, history of radiotherapy in the nervous system, tumor grades, localization, and number of tumors. The history of local and systemic treatments was collected as well, including the type of surgery (total resection or partial resection) and radiotherapy (standard or Cyberknife). Adverse events were recorded as documented by oncologists in the medical chart.

### 2.3. End Points

The primary objective was to identify unfavorable prognostic factors in refractory meningioma. Group 2, refractory to local treatments, was compared to Group 1 to highlight prognostic indicators for early identification of patients who were likely to experience unfavorable outcomes. Furthermore, patient age and sex, tumor localization and grade, number of lesions, and progression-free survival after first-line (PFS-1) and second-line treatment (PFS-2) were compared between the two groups.

The secondary objectives included documenting the clinical progression of patients in Group 2 receiving systemic treatments, with a focus on treatment duration and sequencing, overall survival, six-month progression-free survival (PFS-6 months), median progression-free survival (mPFS), and the associated adverse effects of grade 2 or higher. 

Given the diversity of systemic treatments and the limited number of patients, our study focused on the PFS and OS in patients treated with Bevacizumab and those receiving non-Bevacizumab therapies to provide a unified analysis. Details on the clinical evolution of patients treated with Bevacizumab and Hydroxyurea are presented, as these were the two most frequently administered systemic treatments in our study.

Progression-free survival (PFS) was defined as the time from the initiation of systemic treatment to the date of radiologically confirmed progression or death from any cause. Patients without progression at the last follow-up were censored at that date.

### 2.4. Statistical Analysis

Statistical analyses were performed using IBM SPSS Statistics version 29.0.1.0 and GraphPad Prism version 10.6.0. Variables were assessed using Fisher’s exact tests and Student’s *t*-tests. Survival analysis included Kaplan–Meier curves, with group comparisons made using log-rank tests. Hazard ratios were calculated using univariate cox regression analyses.

## 3. Results

### 3.1. Patient Selection, Clinical and Tumoral Characteristics

**Figure 1:**
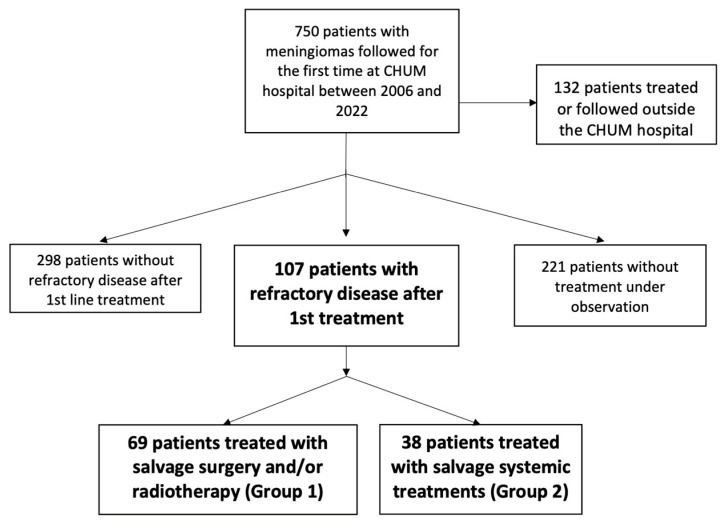
Patient flow diagram illustrating the selection of patients.

Between 2006 and 2022, 750 patients with meningioma were referred to the CHUM Hospital in which 107 patients (14%) progressed after first-line treatment: 69 patients (64%) were treated with salvage surgery and/or radiotherapy (Group 1), and 38 patients (36%) received additional salvage systemic treatments after being refractory to local treatments (Group 2) (Figure 1). The median follow-up time from diagnosis was 7.51 years. 

Table 1 presents the demographic and tumor characteristics of patients in Group 1 and Group 2, followed by Table 2, which presents mortality hazard ratios based on patient characteristics.

Group 2 had a higher proportion of male patients compared to Group 1 (57.2 vs. 33.3%, *p* = 0.024). However, sex did not have a significant statistical impact on survival (HR = 1.062, *p* = 0.879).

The median patient age was 58 years, with the majority (33.6%) in both groups being under 65 years old. When analyzing Kaplan–Meier Curves according to age, patients aged ≥ 65 years had a 10-year survival rate of 53.9%, compared to 82.8% for those under 65. The mortality hazard ratio for age ≥ 65 was statistically significant (HR = 2.820, *p* = 0.009).

Tumors in Group 2 were more frequently classified as grade 2 (52.6% vs. 26.1%) and grade 3 (15.3% vs. 4.3%) compared to Group 1 (*p* = 0.002). Pathologic reports were unavailable for 13 patients: one patient had a presumptive meningioma diagnosis, and 12 others had their brain biopsies performed in another hospital center. Higher tumor grades correlated with poorer survival rates. The 10-year survival rates for patients with grade 1, 2, and 3 were, respectively, 90.5% vs. 52.4% vs. 46.9%. (*p* = 0.004). The mortality hazard ratio for grade 2 and 3 tumors was 4.245 (*p* = 0.004) (Table 2). 

Although most meningiomas were supratentorial (79.4%), Group 2 exhibited a more diverse tumor distribution, including spinal as well as both supra and infratentorial locations (*p* = 0.06). However, tumor location had no significant statistical impact on survival (HR = 0.700, *p* = 0.448). 

Additionally, 80.4% of meningiomas were solitary. The number of meningiomas had no significant statistical impact on survival either (HR = 0.527, *p* = 0.241).

**Figure 2:**
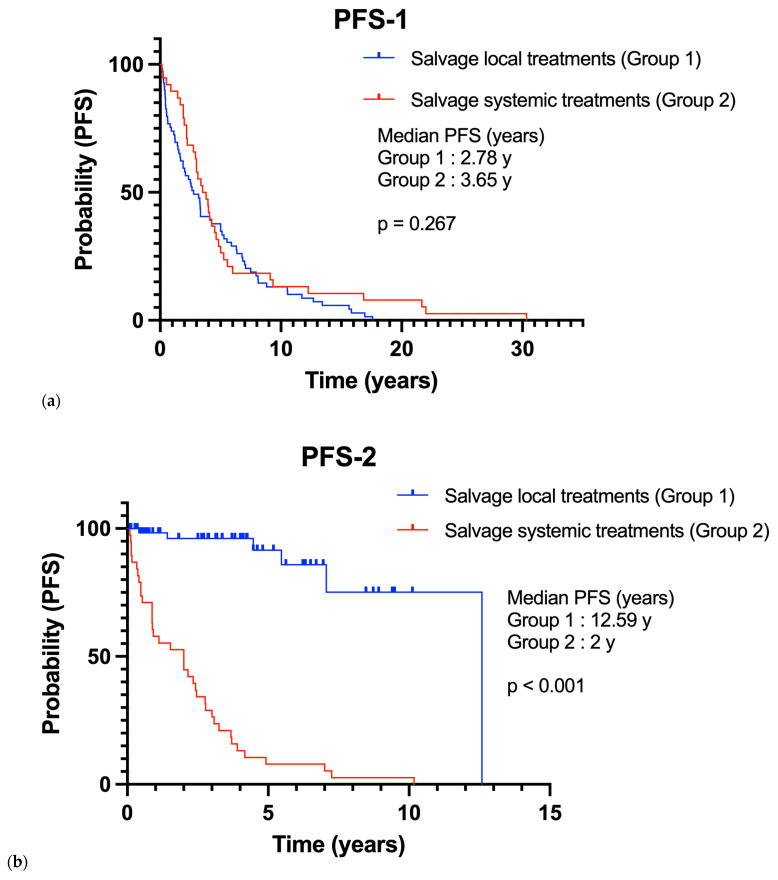
Progression-free survival of Group 1 in comparison with Group 2 (**a**) On first-line therapy (PFS-1); (**b**) On second-line therapy (PFS-2).

Finally, in our recurrent meningioma population, all patients had a first relapse. While all patients in Group 2 also had a second relapse, only 6 (8.7%) patients in Group 1 did. Progression-free survival after first-line treatment (PFS-1) was comparable between Group 1 and Group 2 (mPFS-1: 2.78 vs. 3.65 years; *p* = 0.267) (Figure 2). However, progression-free survival after second-line treatment (PFS-2) was significantly shorter in Group 2 (mPFS-2: 12.59 vs. 2 years; *p* < 0.001) (Figure 2). The presence of disease progression after second-line treatment was associated with a mortality hazard ratio of 4.774 (*p* = 0.004). 

### 3.2. Overall Survival

Patients in Group 1 had significantly better survival compared to those in Group 2. At 10 years, the survival rate was 88.3% for Group 1 versus 67.2% for Group 2 (*p* = 0.009). Among patients whose systemic treatment was discontinued, the mean survival was 8.94 months. 

### 3.3. Local Treatments

Overall, 104 patients had surgery: 68 (98.5%) in Group 1 and 36 (94.7%) in Group 2. Three patients had radiotherapy without surgery: one (1.5%) in Group 1 and two (5.2%) in Group 2. 89 patients had both surgery and radiotherapy: 58 (84%) in Group 1 and 31 (81.6%) in Group 2. 

Total resection was achieved in 57 patients (53.3%) and was similarly distributed between the groups: 36 (52.2%) in Group 1 and 21 (55.3%) in Group 2 (*p* = 0.717). Surgical details were unavailable for 12 patients (11.2) as their procedure was performed in another center. Total resection of the tumor did not have a significant impact on survival (HR = 0.846, *p* = 0.702).

In patients that had radiotherapy, 66 (69.5%) had standard radiotherapy, 13 (13.6%) had Cyberknife, and 15 (15.8%) had both procedures (Table A1). 

### 3.4. Systemic Treatments

**Figure 3:**
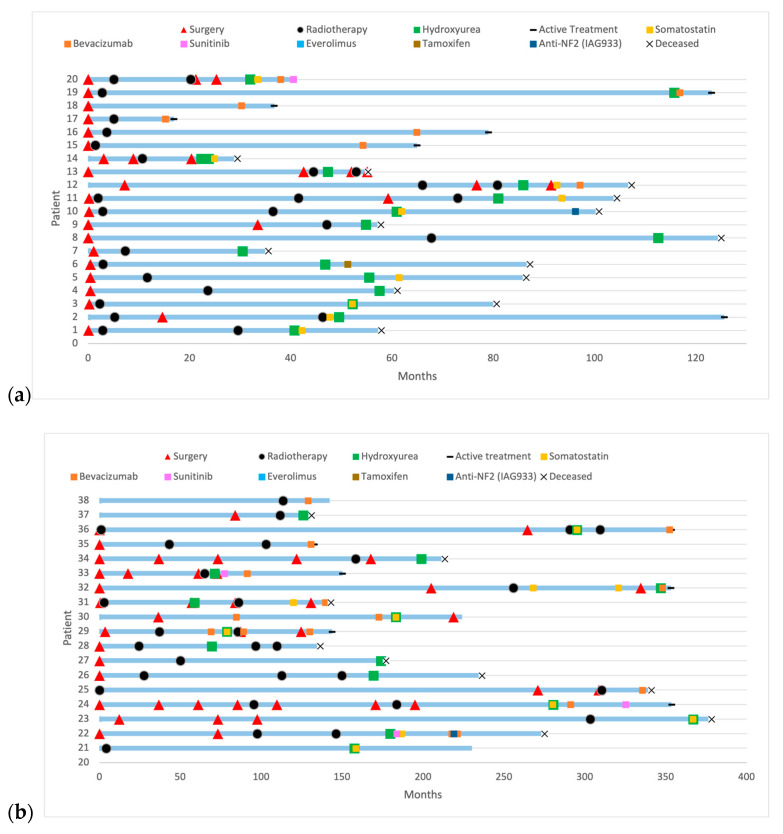
History of patients with refractory meningioma under systemic treatments: (**a**) with a follow-up under 10 years; (**b**) with a follow-up over 10 years.

**Figure 4:**
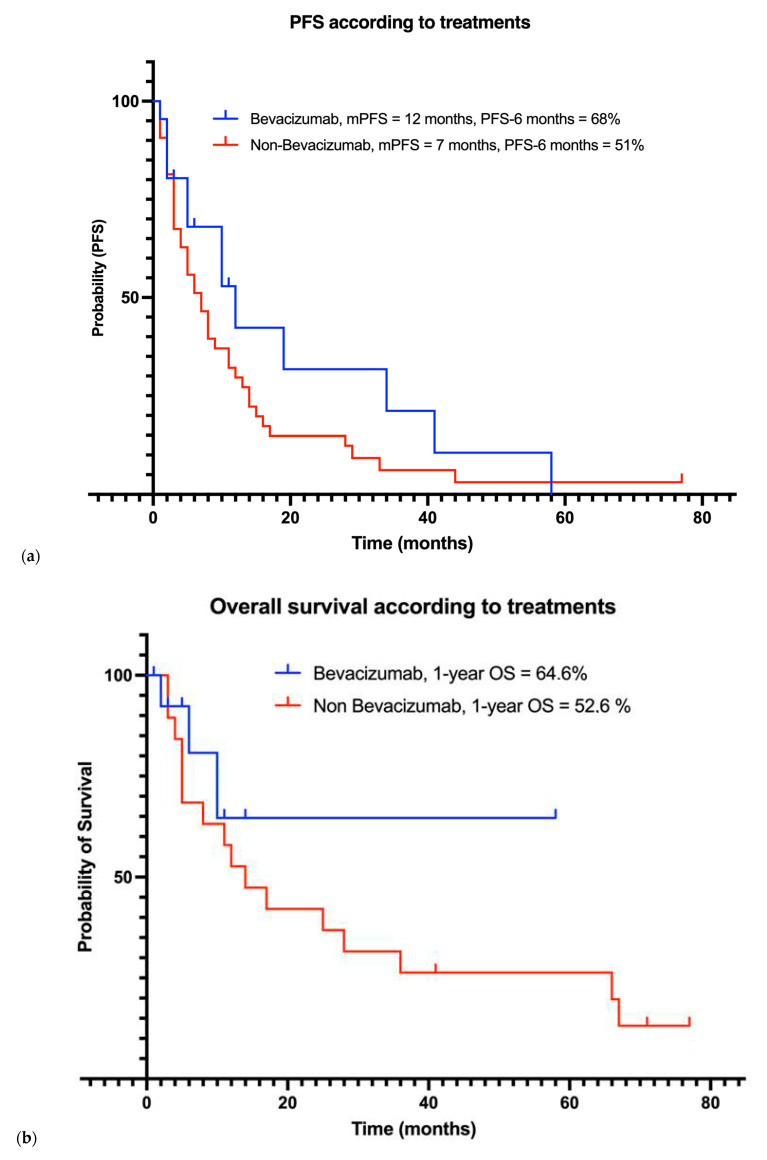
Outcomes of patients under systemic treatments including (**a**) progression-free survival and (**b**) overall survival curves based on systemic treatment received.

38 patients received systemic treatments. Patients received multiple successive local and systemic treatments. Figure 3 illustrates the evolution of patients under systemic treatments over time. Figure 4 includes progression-free survival of each treatment and overall survival according to treatments: Bevacizumab versus non-Bevacizumab. Overall survival was only calculated for treatments that were the last treatment given to at least three patients. Patients under Sunitinib and Somatostatin were not included in the overall survival analysis, as fewer than three patients received these treatments as their last therapy. 

#### 3.4.1. Bevacizumab

A total of 18 patients (47%) received Bevacizumab, which was associated with a median progression-free survival of 12 months (range: 2–58), and a PFS-6 months rate of 68%. The 1-year overall survival (1-year OS) rate was 64.6%. Mean overall survival with treatment was 39.9 (22.59–57.28) months. Nine patients had received prior systemic treatments before starting Bevacizumab. 

At the latest follow-up, 9 patients had stable disease (SD), with a median treatment duration of 3 months. One patient had a minor response and has been on treatment for 6 months. Eight patients had progressive disease (PD), with a median treatment duration of 7 months. Bevacizumab was generally well tolerated, with reported adverse events including one case of proteinuria, one case of hypertension, and a single episode of gastrointestinal bleeding attributed to hemorrhoids.

#### 3.4.2. Non-Bevacizumab Systemic Treatments 

Non-Bevacizumab systemic treatments included Hydroxyurea (*n* = 25, 66%), Somatostatin (*n* = 8, 21%), combination of Hydroxyurea and Somatostatin (*n* = 9, 24%), Everolimus with Sandostatin ( *n* = 1, 3%), Tamoxifen (*n* = 1, 3%), Sunitinib (*n* = 3, 8%), and Anti-NF2 therapy (*n* = 2, 5%). mPFS was 7 months (range 1–44), and the PFS-6 months rate was 51% for these patients. The 1-year OS rate was 52.6%. Mean overall survival for treatment was 14 (3–67) months.

Concerning Hydroxyurea, a total of 25 patients (66%) received Hydroxyurea, of whom 10 did not receive any subsequent treatments. The PFS-6 months was 44.4%, while the 2-year OS rate was 33.3%. Mean overall survival for treatment with Hydroxyurea was 20.67 (6.81–7.31) months. None of the patients who received Hydroxyurea as their only systemic therapy survived. The median treatment duration was 6 months. Adverse events included anemia, thrombocytopenia, neutropenia, and mucositis, each occurring in 3 patients. 

## 4. Discussion

This retrospective study includes patients diagnosed with recurrent meningioma since 2006 and provides valuable insight into the evolution of patient management over the past 19 years, including the sequence of treatments received and changes in clinical practice. With a median follow-up of 7.5 years, it is the only retrospective study to evaluate multiple treatment strategies for recurrent meningioma over such an extended period. 

The overall survival of patients with salvage local treatments (Group 1) was similar compared to non-recurrent meningioma from CBTRUS data (10-year OS: 83.7% vs. 83.2%). Thus, the presence of a first relapse does not change patient prognosis in this cohort. Survival was lower for patients in Group 2, representing those patients in whom local treatments failed after the first recurrence and who received systemic treatments (10-year OS: 63.9% vs. 83.2%). The second relapse occurred in all patients, and the PFS-2 was shorter in Group 2 compared to Group 1. Second relapse emerged as a new key predictor of worse survival (HR = 4.77), undescribed in the past. This emphasizes the unmet need to identify systemic treatments for this relapsing population with no local treatment options [1]. 

Other prognostic factors like age ≥ 65 years (HR = 2.82) and tumor grade 2 or 3 (HR = 4.25) were confirmed by our study as significant prognostic factors, consistent with prior studies [1,4]. 

Furthermore, our data highlight Bevacizumab as a reasonable systemic treatment with a PFS-6 months rate achieving 68% and mPFS 12 (2–58) months in 18 unselected patients. This mPFS is shorter than the mPFS of 22 months reported in a Phase II prospective trial involving 42 selected patients [8,9,10]. However, our population was heavily pretreated, having undergone multiple successive treatments before receiving Bevacizumab. Encouraged by the minor responses observed in these patients, these findings reinforce the potential therapeutic role of Bevacizumab, leading to its increased use in our institution over time. 

On the other hand, the use of Hydroxyurea in our institution decreased over time due to its limited efficacy, with an mPFS of 4 months, which is consistent with the literature (mPFS: 2 months) [11,12]. 

Among other systemic treatments reported in the literature, we limited our analysis of PFS and OS to Bevacizumab versus non-Bevacizumab options, given the small number of patients in our study. Our results reported a PFS-6 months of 51% in non-Bevacizumab treatments. In prospective trials, PFS-6 rates of 44.4%, 42%, and 55% have been observed with long-acting somatostatin analogues [13], Sunitinib [14], and the combination of Everolimus and Octreotide [15], respectively, in recurrent meningioma. Bevacizumab has shown higher rates of disease control in our study. However, larger prospective studies are required to enable robust head-to-head comparisons.

Systemic treatments were generally well tolerated in our study. Grade 2 or higher events were observed in three patients receiving Bevacizumab, including hypertension and proteinuria, which have been documented in the literature [8]. For Hydroxyurea, cytopenias were also reported, as expected based on prior studies [11]. 

It is important to note that among patients whose systemic treatment was discontinued, the mean survival was 8.94 months. While meningiomas are often perceived as a benign disease, our results highlight the potentially fatal nature of recurrent meningiomas. There is an urgent need for systemic treatments that can improve survival in the most aggressive cases.

Our study has certain limitations due to its inherent retrospective nature. Meningiomas of grades 1, 2, and 3 were included in the analysis despite their different biological behavior. The small number of patients prevented a reliable sub-analysis according to tumor grades. Nevertheless, our study reflects real-world practice, where some lower-grade tumors may evolve into higher-grade lesions at recurrence without systematic pathological reassessment. The heterogeneity of meningiomas, therefore, remains an inherent challenge. Also, radiologic follow-ups were not standardized. Treatment choices were influenced by the evolution of the literature since 2006, the accessibility within the province’s public health system, and private coverage of patients, which explains the different treatments given. Lastly, the small number of patients limits the statistical power to differentiate the efficacy of treatments. 

However, our study includes long-term follow-up with detailed clinical data collection as it compares different treatments and sequences, including their associated adverse effects. Additionally, our non-selected patient population better reflects real-world clinical practice. As a single-center study, the standard of care and clinical practice were relatively uniform among physicians. Our results identified prognostic factors that can help clinicians detect aggressive cases earlier. This study supports Bevacizumab as a reasonable option in cases of local treatment failure in recurrent meningioma. Preliminary findings in 2024 influenced our practice, leading us to prioritize Bevacizumab in treatment decisions. However, its cost and limited availability may restrict its use in some countries, underscoring the need to develop additional systemic treatments for recurrent meningioma. 

Since 2021, molecular profiling has been incorporated into meningioma diagnosis [16]. As our study began in 2006, next-generation sequencing (NGS) was not available for patients. Molecular and genomic profiling have shown promising results in predicting tumor response to radiotherapy and aiding physicians in surgical and radiotherapy decision-making [17]. Integrating NGS with the prognostic factors identified in our study could further help with the earlier identification of patients who will progress and the implication of these patients in prospective studies.

## Figures and Tables

**Table 1:** Demographic and tumor characteristics of patients.

Characteristics		Total (107, %)	Group 1 (69, %)	Group 2 (38, %)	*p*-Value
Sex	Male	45 (42.1)	23 (33.3)	22 (57.2)	0.024
Age	≥65 years old	36 (33.6)	25 (36.2)	11 (28.9)	0.524
	Median	58	59	54	
History of brain or spinal radiotherapy	Yes	3 (2.8)	0	3 (7.9)	0.043
NF2 Mutation	Yes	6 (5.6)	0	6 (15.8)	<0.001
	Somatic	2 (1.9)	0	2 (5.3)	0.124
Tumor grade	1	47 (43.9)	38 (55.1)	9 (23.7)	0.002
	2	38 (35.5)	18 (26.1)	20 (52.6)	
	3	9 (8.4)	3 (4.3)	6 (15.3)	
	NA	13 (12.1)	10 (14.5)	3 (7.9)	
Localization of tumor	Supratentorial	85 (79.4)	59 (85.5)	26 (68.4)	0.060
	Infratentorial	14 (13.1)	8 (11.6)	6 (15.8)	
	Supra and infratentorial	6 (5.6)	1 (1.4)	5 (13.2)	
	Spinal	2 (1.9)	1 (1.4)	1 (2.6)	
Number of tumors	Unique	86 (80.4)	56 (81.2)	30 (78.9)	0.803
Type of surgery—Extent of resection	Total resection	57 (53.3)	36 (52.2)	21 (55.3)	0.717
	NA	12 (11.2)	8 (11.5)	4 (10.5)	

**Table 2:** Hazard ratios for mortality based on patient age, sex, presence of second progression, tumor grade, localization, and number of tumors.

Variables	Hazard Mortality Ratio	*p*-Value
Grade 2 or 3	4.25 (1.579–11.411)	0.004
Age ≥ 65 y-o	2.82 (1.303–6.106)	0.009
Presence of 2nd progression	4.77 (1.627–14.012)	0.004
Localization	0.70 (0.278–1.759)	0.448
Number	0.53 (0.180–1.539)	0.241
Sex	1.06 (0.490–2.300)	0.879
Type of surgery—extent of resection	0.846 (0.359–1.995)	0.702

## Data Availability

The data presented in this study are available upon request from the corresponding author due to privacy and ethical reasons.

## Abbreviations

The following abbreviations are used in this manuscript:

PFS-6	6-month progression-free survival
mPFS	Median progression-free survival
PFS-1	Progression-free survival on first-line therapy
PFS-2	Progression-free survival on second-line therapy
OS	Overall survival
HR	Hazard ratio

## Appendix A

**Table A1:** Local treatments received in Group 1 and Group 2.

Local Treatment		Group 1 (69, %)	Group 2 (38, %)	Total (107, %)
**Surgery**		68 (98.6)	36 (94.7)	104 (97.2)
	Total	36 (52.2)	21 (55.3)	57 (53.3)
	Partial	21 (30.4)	10 (26.3)	31 (28.9)
	Both	3 (4.3)	1 (2.6)	4 (3.7)
	ND	8 (11.5)	4 (10.5)	12 (11.2)
Surgery + Radiotherapy		58 (82.9)	31 (81.6)	89 (83)
Radiotherapy without surgery		1 (1.5)	2 (5.2)	3 (2.8)
Types of radiotherapy		59 (84)	36 (96.5)	95 (88.7)
	Standard radiotherapy	46 (77.9)	21 (55.3)	66 (61.7)
	Cyberknife	10 (17)	3 (7.9)	13 (12.1)
	Combined	3 (5)	12 (31.6)	15 (14)
